# Diagnostic value of metagenomic next-generation sequencing in sepsis and bloodstream infection

**DOI:** 10.3389/fcimb.2023.1117987

**Published:** 2023-02-10

**Authors:** Cuihong Qin, Shuguang Zhang, Yingying Zhao, Xianfei Ding, Fei Yang, Yangchao Zhao

**Affiliations:** ^1^ General ICU, Henan Key Laboratory of Critical Care Medicine, Henan Engineering Research Center for Critical Care Medicine, Zhengzhou Key Laboratory of Sepsis, The First Affiliated Hospital of Zhengzhou University, Zhengzhou, China; ^2^ Cardiopulmonary Support Center, The First Affiliated Hospital of Zhengzhou University, Zhengzhou, China

**Keywords:** bloodstream infection, sepsis, blood culture, MNGs, diagnosis, Intensive Care Unit

## Abstract

**Objective:**

To evaluate the diagnostic value of metagenomic next-generation sequencing (mNGS) in sepsis and bloodstream infection (BSI).

**Methods:**

A retrospective analysis of patients diagnosed with sepsis and BSI at the First Affiliated Hospital of Zhengzhou University from January 2020 to February 2022 was conducted. All the patients underwent blood culture and were divided into mNGS group and non-mNGS group according to whether mNGS was performed or not. The mNGS group was further divided into early group (< 1 day), intermediate group (1–3 days), and late group (> 3 days) according to the time of mNGS inspection.

**Results:**

In 194 patients with sepsis and BSI, the positive rate of mNGS for identifying pathogens was significantly higher than that of blood culture (77.7% vs. 47.9%), and the detection period was shorter (1.41 ± 1.01 days vs. 4.82 ± 0.73 days); the difference was statistically significant (*p* < 0.05). The 28-day mortality rate of the mNGS group (*n* = 112) was significantly lower than that of the non-mNGS group (*n* = 82) (47.32% vs. 62.20%, *p* = 0.043). The total hospitalization time for the mNGS group was longer than that for the non-mNGS group (18 (9, 33) days vs. 13 (6, 23) days, *p* = 0.005). There was no significant difference in the ICU hospitalization time, mechanical ventilation time, vasoactive drug use time, and 90-day mortality between the two groups (*p* > 0.05). Sub-group analysis of patients in the mNGS group showed that the total hospitalization time and the ICU hospitalization time in the late group were longer than those in the early group (30 (18, 43) days vs. 10 (6, 26) days, 17 (6, 31) days vs. 6 (2, 10) days), and the ICU hospitalization time in the intermediate group was longer than that in the early group (6 (3, 15) days vs. 6 (2, 10) days); the differences were statistically significant (*p* < 0.05). The 28-day mortality rate of the early group was higher than that of the late group (70.21% vs. 30.00%), and the difference was statistically significant (*p* = 0.001).

**Conclusions:**

mNGS has the advantages of a short detection period and a high positive rate in the diagnosis of pathogens causing BSI and, eventually, sepsis. Routine blood culture combined with mNGS can significantly reduce the mortality of septic patients with BSI. Early detection using mNGS can shorten the total hospitalization time and the ICU hospitalization time of patients with sepsis and BSI.

## 1 Introduction

Bloodstream infection (BSI) refers to positive blood culture in patients with signs and symptoms of systemic infection, which can be classified as primary, that is, not secondary to an identified infection, or secondary, that is, following a localized infectious disease. As a common life-threatening disease in the intensive care unit (ICU), severe cases of BSI can lead to sepsis and septic shock, with high mortality rates (Massart et al., 2021; Guzek et al., 2022). Early and effective antibiotic course is the key to treating BSI. If the pathogen cannot be identified early in the disease, effective antibiotics will be administered late, which often leads to poor clinical outcomes (Lindberg et al., 2020). In severe sepsis and septic shock, the survival rate of patients decreases by an average of 7.6% every hour of delayed use of effective antibiotics. If proper antibiotic treatment is not given within 1 hours, the survival rate of sepsis can decrease (Andersson et al., 2019; Sanguanwit et al., 2022). The current guidelines recommend that antibiotic treatment be started as early as 1 hour after diagnosing sepsis or septic shock (Evans et al., 2021). However, in actual clinical work, about 46% of empirical antibiotic treatment is proven inappropriate, leading to an increase in mortality and antibiotic resistance risk, and toxic reactions (Campion and Scully, 2018). Accurate identification of BSI pathogens is of great value for early guidance of antibiotic treatment, better antibiotic management, and improved clinical outcomes.

At present, blood culture is still the gold standard for BSI diagnosis. Expert consensus on bloodstream infection in critically ill patients recommends that at least two sets of aerobic and anaerobic cultures be sampled after skin disinfection following strict aseptic guidelines (Timsit et al., 2020). However, prolonged blood culture tests, low microbial inoculum, or antibiotics before blood draw will reduce the positive rate of blood culture (Pilecky et al., 2019; Rand et al., 2019; Yu et al., 2020). In recent years, metagenomic next-generation sequencing (mNGS) has become a new method for detecting pathogenic microorganisms. It can analyze whole microbial colonies in clinical samples without needing prolonged culture (Miao et al., 2018; Blauwkamp et al., 2019). It has been reported that mNGS can help identify pathogenic microorganisms of suspected BSI patients with high sensitivity and specificity (Grumaz et al., 2019). However, few studies on the clinical application of mNGS in BSI have been carried out, most of which are case reports or small sample studies (Sun et al., 2022). Therefore, it is urgent to evaluate the importance of the mNGS technique in the diagnosis of sepsis and BSI.

## 2 Materials and methods

### 2.1 Study subjects

This study is a retrospective analysis of septic patients with BSI from the First Affiliated Hospital of Zhengzhou University. No blood tests or drug interventions were carried out. Electronic medical records of patients were reviewed to collect clinical data and laboratory results. This study was approved by the Ethics Committee of the First Affiliated Hospital of Zhengzhou University, with the ethics number 2022-KY-0225-002.

### 2.2 Research methods

#### 2.2.1 Inclusion criteria

Septic patients with bloodstream infection admitted to the First Affiliated Hospital of Zhengzhou University, China, from January 2020 to February 2022, were screened for this study. According to the sepsis 3.0 standard (Singer et al., 2016), sepsis can be diagnosed if the following two conditions are met: Definite or suspected infection exists; sequential organ failure assessment (SOFA) score increased by ≥2 points compared with baseline. Organ dysfunction was assessed by the SOFA scoring system, and disease severity was assessed by the acute physiology and chronic health assessment II (APACHE II) scoring system. Referring to the 2016 US CDC clinical diagnostic criteria for bloodstream infection, BSI can be defined as: Fever (T ≥ 38.0°C), chills, or accompanied by hemodynamic instability that cannot be explained by infection in a specific body part (CDC, 2016). All patients had their blood taken for culture after admission.

#### 2.2.2 Exclusion criteria

No blood culture was performed after admission; The hospitalization time was less than 24 hours; Age < 18 years old; At the terminal stage of malignant diseases such as decompensated cirrhosis and malignant tumor, the expected survival time was no more than 3 months; Missed follow-up.

### 2.3 Analysis of information collected

The clinical data collection of the selected subjects included: age, gender, ICU hospitalization time, total hospitalization time, mechanical ventilation time and vasoactive drug use time, past medical history, laboratory results within 24 hours after admission (white blood cell, red blood cell, platelet, hemoglobin, serum creatinine, urea nitrogen, alanine aminotransferase, glutamic oxaloacetic transaminase, total bilirubin, albumin, plasma prothrombin time, activated partial thromboplastin time, and international standardized ratio), blood culture results (pathogen type, submission time, and detection time), mNGS results (pathogen type, sequence number, relative abundance, submission time, and detection time), serum infection markers (erythrocyte sedimentation rate, C-reactive protein, interleukin-6, and procalcitonin), ICU special treatment data (based on antibiotic adjustment (blood culture, mNGS or clinical)), adjustment contents, adjustment time, APACHE II and SOFA scores within 48 hours of admission, and APACHE II and SOFA scores 7 days after antibiotic adjustment.

### 2.4 Follow-up and grouping

Telephone follow-up was conducted to document patient outcome indicators. The primary outcome was determined by 28-day mortality. The secondary outcome was to determine the ICU hospitalization time, total hospitalization time, mechanical ventilation time, vasoactive drug use time, and 90-day mortality. The patients were divided into mNGS group and non-mNGS group according to whether they received mNGS or not, respectively. The patients in the mNGS group were further divided into early group (< 1 day), intermediate group (1–3 days), and late group (> 3 days) according to the application time of mNGS.

### 2.5 Statistical analysis

SPSS 22.0 software was used for data analysis. The Kolmogorov-Smirnov test was used to verify the normality of the measurement data. The data in accordance with the normal distribution were described by the mean and standard deviation (SD). The comparison between groups adopted the T-test or corrected T-test according to the homogeneity of variance. The median (quartile) [M (QL, QU)] was used to describe the distribution characteristics of the non-normal distribution, and the Mann-Whitney U nonparametric test was used for comparison between groups. The Chi-square test was used to compare variables. *p* < 0.05 was considered statistically significant.

## 3 Results

### 3.1 Patient baseline characteristics

**Figure 1:**
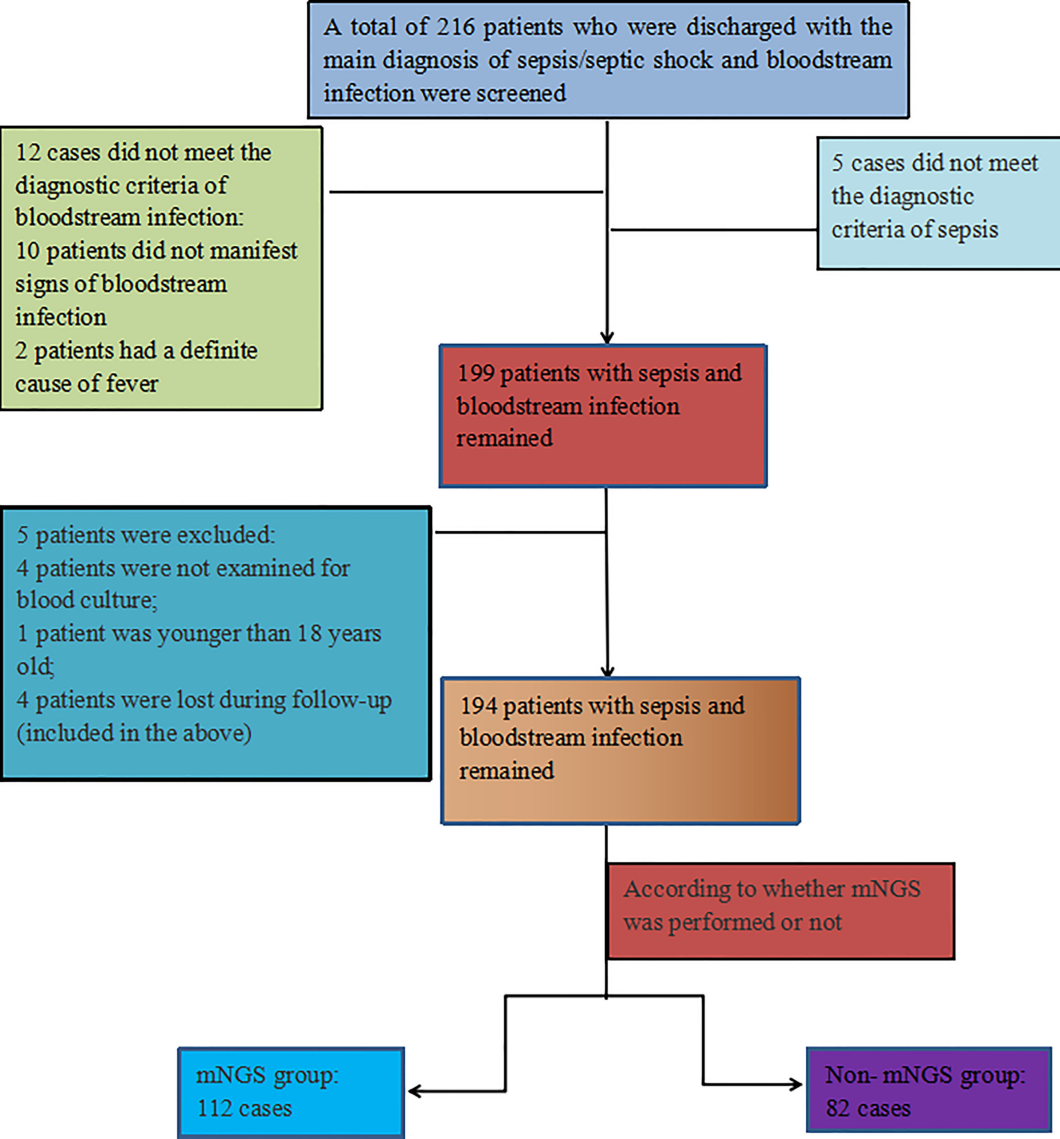
Inclusion process chart.

A total of 216 discharged patients diagnosed with sepsis and bloodstream infection were screened for this study, of which 12 patients did not meet the diagnostic criteria of bloodstream infection, 5 patients did not meet the diagnostic criteria of sepsis, and 4 patients had no blood culture, 1patient was not age-compatible and 4 patients were lost during follow-up(included in the above), resulting in 194 patients with sepsis and BSI remaining in this study, including 128 males (66.0%) and 66 females (34.0%). 112 cases were in the mNGS group (blood culture and mNGS were performed), and 82 cases were in the non-mNGS group (blood culture only). The positive rate of mNGS was 77.7% (87/112), and that of blood culture was 47.9% (93/194). **Figure 1** illustrates the inclusion process.

The mean age of the patients was 55.61 ± 15.87 years, and SOFA and APACHE II scores were 8.37 ± 4.67 and 19.27 ± 8.60, respectively. 109 patients died within 28 days, and the 28-day mortality rate was 56.2%; 135 patients (69.6%) received mechanical ventilation, 128 patients (70.0%) received vasoactive drugs, and 80 patients (41.2%) received antibiotics before blood culture.

The average period of mNGS detection was 1.41 ± 1.01 days, and the average time of traditional blood culture detection was 4.82 ± 0.73 days. The time of mNGS detection was significantly shorter than that of traditional blood culture detection (*p* < 0.05).

### 3.2 Detection of pathogens in blood culture

**Figure 2:**
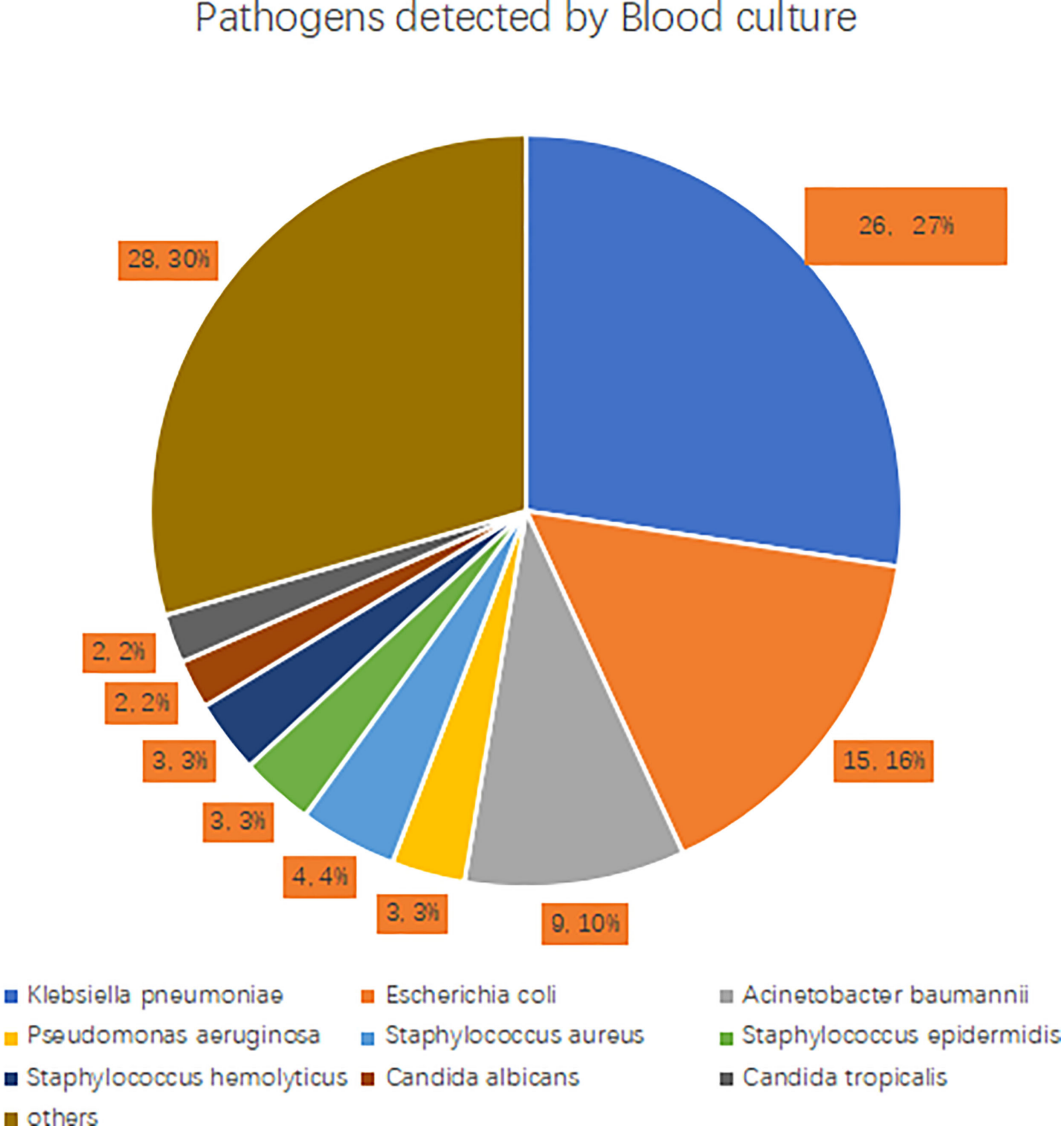
Pathogens detected by blood culture.

Among the 194 patients, 93 (47.9%) had a positive blood culture, including 65 cases of Gram-negative bacteria, 22 cases of Gram-positive bacteria, and 6 cases of fungi. Among the Gram-negative bacteria, 26 cases were *Klebsiella pneumoniae* (40.0%), 15 cases were *Escherichia coli* (23.1%), 9 cases were *Acinetobacter baumannii* (13.8%), and 3 cases were *Pseudomonas aeruginosa* (4.6%). Gram-positive bacteria strains were mainly *Staphylococcus aureus* (4 cases, 18.2%), *Staphylococcus epidermidis* (3 cases, 13.6%), and *Staphylococcus haemolyticus* (3 cases, 13.6%). The most common fungi detected were *Candida albicans* (2 cases, 33.3%) and *Candida tropicalis* (2 cases, 33.3%). See **Figure 2** for details.

### 3.3 Pathogens detected by mNGS

**Figure 3:**
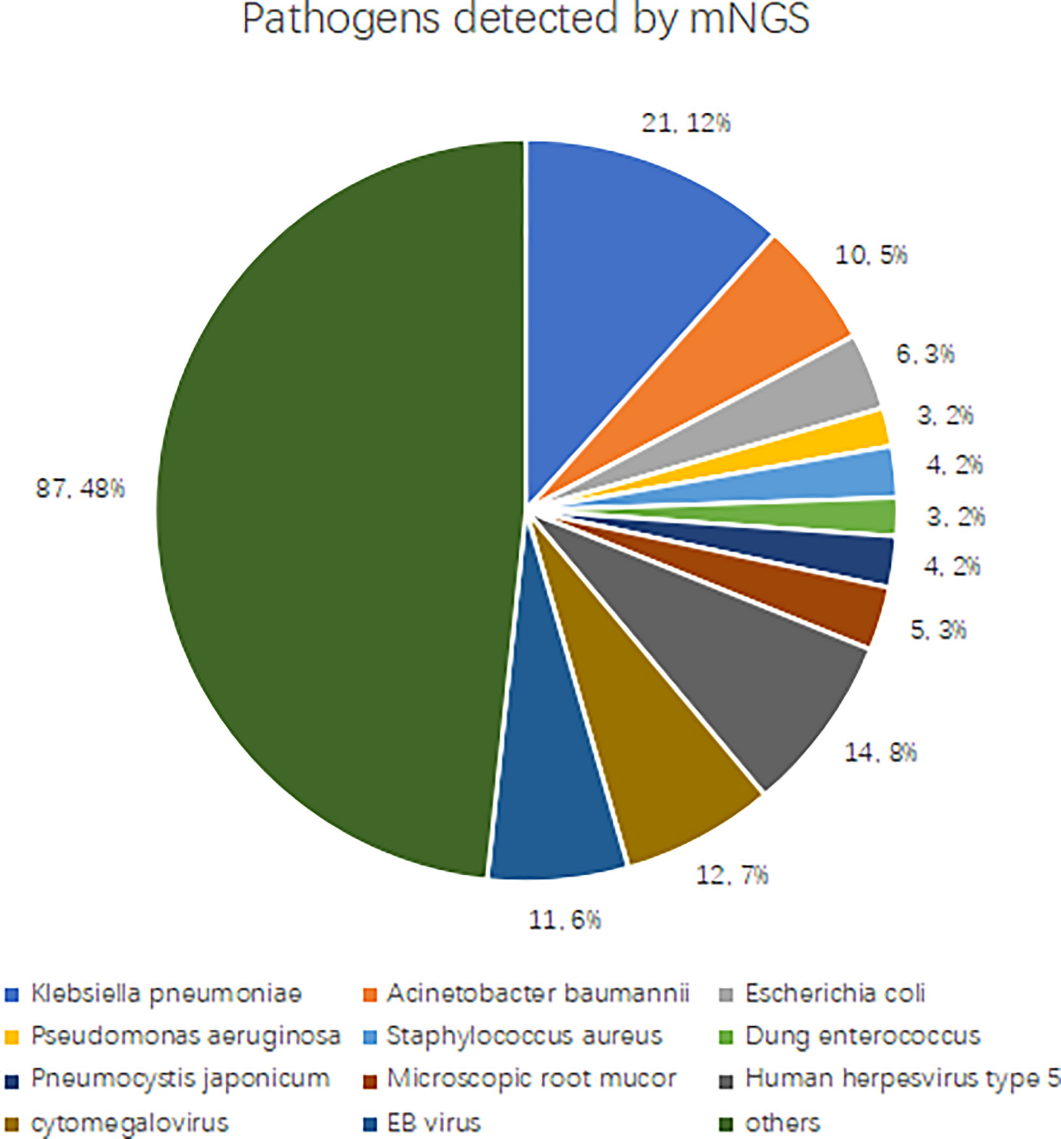
Pathogens detected by mNGS.

We determined pathogens according to the sequence number and relative abundance of detected pathogens in combination with clinical practices. If several pathogens are detected in a blood sample by mNGS, a maximum number of four pathogens with high sequence numbers and relative abundance are clinically suspected. mNGS detected 180 types of suspected pathogens, and the most commonly detected Gram-negative bacteria were *Klebsiella pneumoniae* (*n* = 21), *Acinetobacter baumannii* (*n* = 10), *Escherichia coli* (*n* = 6), and *Pseudomonas aeruginosa* (*n* = 3). The most commonly detected Gram-positive bacteria were *Staphylococcus aureus* (*n* = 4), and *Enterococcus faecalis* (*n* = 3). The most frequently detected fungi were *Pneumocystis Yersinia* (*n* = 4) and *Rhizopus macrocarpa* (*n* = 5). The most commonly detected viruses were human herpesvirus type 5 (*n* = 14), cytomegalovirus (*n* = 12), and Epstein-Barr virus (*n* = 11). See **Figure 3** for details.

### 3.4 Baseline data

The difference in gender, age, APACHE II and SOFA scores 48h after admission between the mNGS group and the non-mNGS group was not significant (*p* > 0.05). In the non-mNGS group, the proportion of stroke patients was significantly higher, and hemoglobin was significantly lower compared to the mNGS group (*p* < 0.05). See **Table S1** for details.

### 3.5 Comparison of primary and secondary outcomes between mNGS group and non-mNGS group

The 28-day mortality rate of the mNGS group was significantly lower than that of the non-mNGS group (*p* = 0.043). The total hospitalization time of the mNGS group was significantly longer than that of the non-mNGS group (*p* = 0.005). There was no significant difference in the ICU hospitalization time, mechanical ventilation time, vasoactive drug use time, and 90-day mortality between the two groups (*p* > 0.05). See **Table 1** for details.

**Table 1:** Comparison of primary and secondary outcomes between mNGS group and non-mNGS group.

	mNGS group (*n* = 112)	Non-mNGS group (*n* = 82)	*p*
28-day mortality	53 (47.32%)	51 (62.20%)	0.043
Total hospitalization time (d)	18 (9, 33)	13 (6, 23)	0.005
ICU hospitalization time (d)	7 (3, 15.75)	6.5 (3, 15.50)	0.355
Mechanical ventilation time (h)	29.5 (0, 193.50)	46 (0, 144)	0.709
Vasoactive drug use time (h)	27 (0, 142.5)	36 (0, 120)	0.815
90-day mortality	64 (57.14%)	57 (69.51%)	0.099

### 3.6 Consistency analysis of blood culture and mNGS pathogen detection

**Figure 4:**
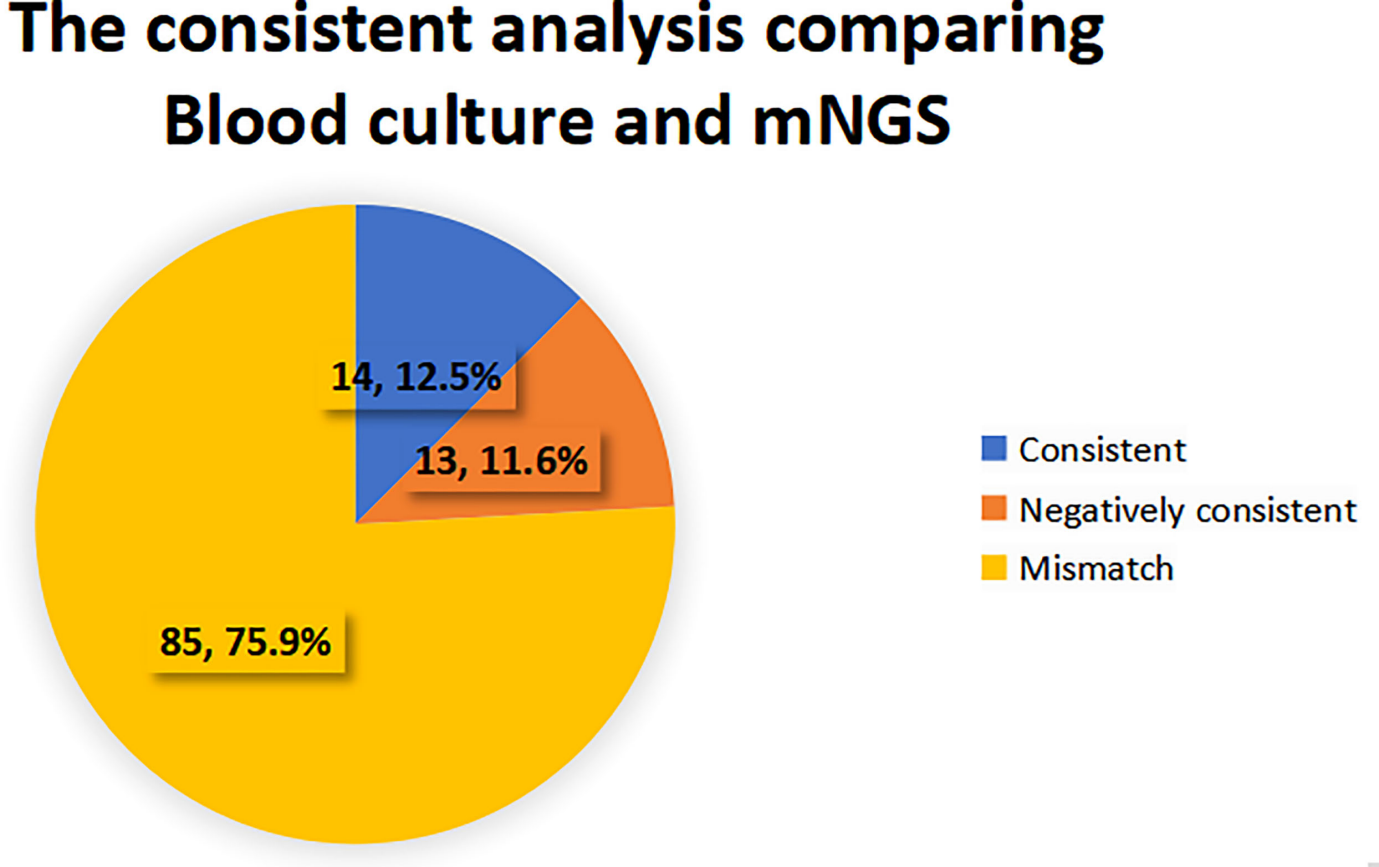
Consistency analysis of pathogens detected by blood culture and mNGS.

**Figure 5:**
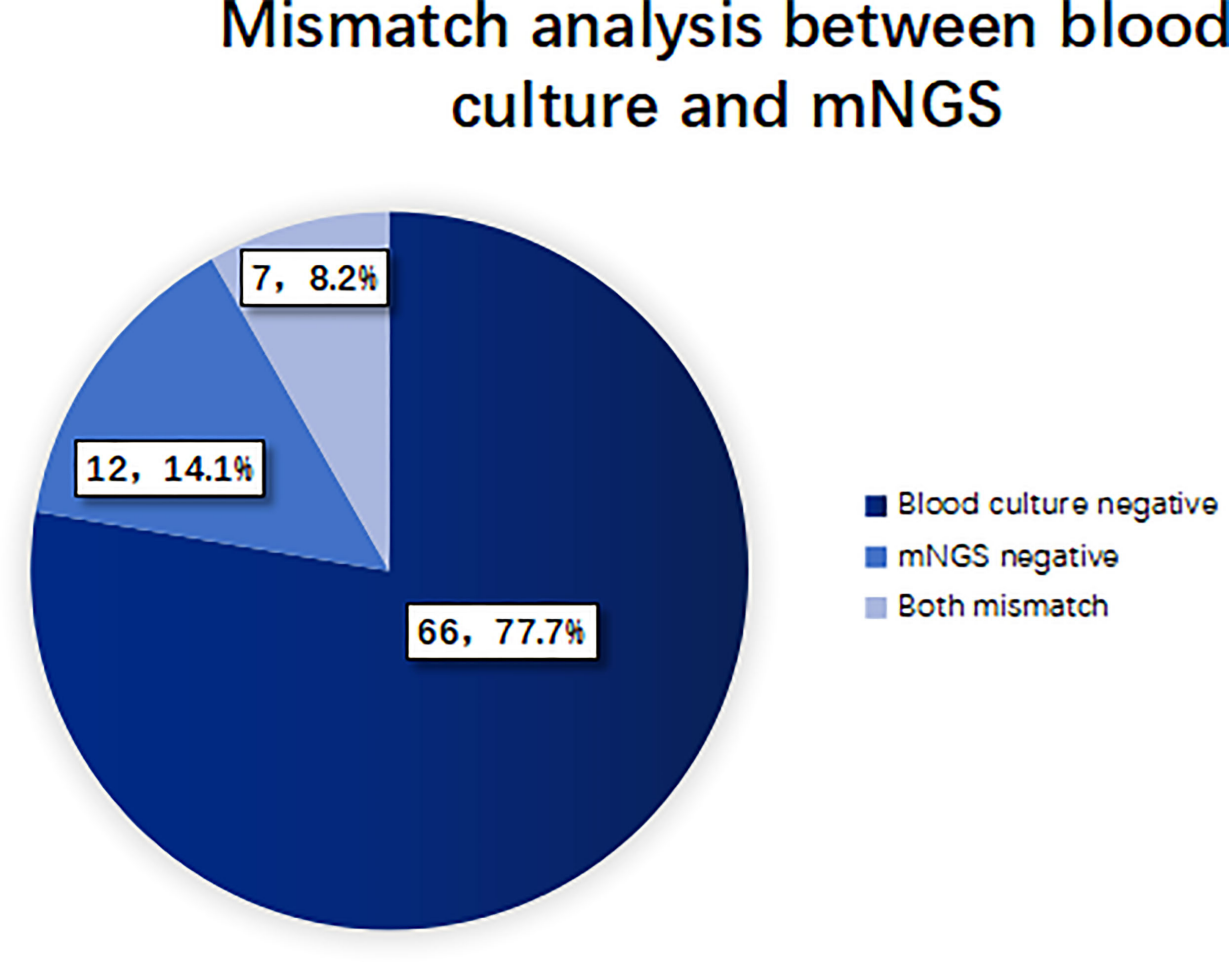
Inconsistency analysis of pathogens detected by blood culture and mNGS.

When the pathogen detected by mNGS is the same as that detected by blood culture, the detection results are considered consistent. If mNGS detects more pathogens than the blood culture method does, and at least one of them is consistent with blood culture, the test results are also considered consistent. When no pathogen is detected by either method, the test results are considered to be negatively consistent. When the pathogens identified by the two methods are completely different or when one method is positive while the other is negative, the test results are considered inconsistent. 27 cases (24.1%) of pathogens detected in mNGS group were consistent with blood culture results, of which 14 cases (12.5%) were positive and 13 cases (11.6%) were negative. 85 cases (75.9%) were inconsistent, of which 66 cases (77.7%) were negative by the blood culture method, 12 cases (14.1%) were negative by the mNGS method, and 7 cases (8.2%) were inconsistent with the pathogens detected by the two methods. See **Figures 4**, **5** for details.

### 3.7 The application of mNGS to guide clinical treatment

The adjustment times of antibiotics in the mNGS group were 2.13 ± 1.15, and those in the non-mNGS group were 1.73 ± 1.40. There was no significant difference between the two groups (*p* > 0.05). 27 patients (13.9%) had their first antibiotic treatment adjusted according to mNGS test results, 15 patients (7.7%) had their first antibiotic treatment adjusted according to blood culture results, and the rest of the patients had their first antibiotic treatment adjusted according to clinical manifestations and overall condition. After the antibiotic regimen was adjusted for the first time, we observed and followed up on the APACHE II and SOFA scores for the next 7 days and found that the difference in APACHE II and SOFA scores between the two groups after 7 days of treatment was not significant (*p* > 0.05). See **Table 2** for details.

**Table 2:** Clinical medication guidance between mNGS group and non-mNGS group.

	mNGS group (*n* = 112)	Non-mNGS group (*n* = 82)	*p*
Adjustment times of antibiotics	2.13 ± 1.15	1.73 ± 1.40	0.060
Antibiotics used before blood culture	51 (45.54%)	29 (35.37%)	0.185
APACHE II score 7 days after the first adjustment of antibiotics	14.60 ± 6.43	16.00 ± 6.35	0.346
SOFA score 7 days after the first adjustment of antibiotics	7.60 ± 4.21	7.07 ± 4.12	0.809

### 3.8 Sub-group analysis: The effect of early, intermediate, and late mNGS on patients

According to the mNGS detection time after admission, patients in the mNGS group were divided into the early group (within one day after enrollment), the intermediate group (1–3 days after enrollment), and the late group (3 days after enrollment). There was no significant difference in baseline data between the three groups (*p* > 0.05; see **Table S2** for details). However, the 28-day mortality, the ICU hospitalization time, and the total hospitalization time were significantly different among the three groups (*p* < 0.05; see **Table 3** for details). There was no significant difference in mechanical ventilation time, vasoactive drug use time, or 90-day mortality among the three groups (*p* > 0.05; see **Table 3** for details).

**Table 3:** Comparison of primary and secondary outcomes in early, intermediate, and late groups.

	Early group (*n* = 47)	Intermediate group (*n* = 35)	Late group (*n* = 30)	*p*
Antibiotics used before blood culture	22 (46.81%)	19 (54.29%)	10 (33.33%)	0.233
Total hospitalization time (d)	10 (6, 26)	19 (10, 34)	30 (18, 43)	0.002
ICU Hospitalization time (d)	6 (2, 10)	6 (3, 15)	17 (6, 31)	0.002
28-day mortality	33 (70.21%)	16 (45.71%)	9 (30.00%)	0.002
90-day mortality	33 (70.21%)	20 (57.14%)	13 (43.33%)	0.063
Mechanical ventilation time (h)	27 (1, 56)	18 (0, 179)	119 (0, 297)	0.666
Vasoactive drug use time (h)	28 (2, 78)	10 (0, 96)	30 (0, 239)	0.647

Pairwise comparison was conducted for the outcome indices with statistical differences among the three groups. The results showed no statistical difference in the total hospitalization time between the early group and the intermediate group (*p* = 0.418). The total hospitalization time in the early group was shorter than that in the late group, and the difference was statistically significant (*p* = 0.001). The ICU hospitalization time in the early group was shorter than in the intermediate group, and the difference was statistically significant (*p* = 0.005). The ICU hospitalization time in the early group was significantly shorter compared to the late group (*p* < 0.001). The difference in the 28-day mortality rate between the early and intermediate groups was not statistically significant (*p* = 0.025), but the 28-day mortality rate of the early group was significantly higher than that of the late group (*p* = 0.001).

## 4 Discussion

The application of mNGS in patients with sepsis and BSI, especially to investigate whether blood culture combined with mNGS can reduce the mortality rate and the clinical implications of early and late mNGS, has rarely been reported. Our study has shown that mNGS has the advantages of a short detection time and a high positive rate in early pathogen diagnosis of BSI and sepsis. Routine blood culture combined with mNGS can reduce the 28-day mortality rate of patients with sepsis and BSI. Early mNGS can shorten the total hospital stay and the ICU hospitalization period.

BSI is a common infectious disease that can eventually lead to severe sepsis and septic shock. It is more likely to occur in patients who have been in ICU for a long time, have had invasive procedures, and are immunosuppressed (Massart et al., 2021). Common pathogens include bacteria, fungi, viruses, and atypical pathogens. For BSIs, broad-spectrum antibiotics or empiric therapy are initially administered before pathogen identification and then adjusted to targeted therapy based on microbiological analysis. Early identification of the pathogen in severe infections resulting in sepsis and septic shock can guide precise treatment (Zhou et al., 2019). Ineffective or inappropriate antibiotic therapy can lead to the generation of multi-drug resistant bacteria, resulting in longer hospital stay, ICU stay, and higher mortality rate (Kumar et al., 2006; Andersson et al., 2019; Raad et al., 2021; Ababneh et al., 2022; Kanj et al., 2022).

Traditional blood culture remains the gold standard for the diagnosis of BSI. However, the positive rate of blood culture is low. It is greatly affected by prior antibiotic use and takes a long time to incubate (Greninger and Naccache, 2019). The detection time of the mNGS method is short, and its application in the diagnosis of pulmonary infection, pediatric BSI, and intracranial infection has been reported in various studies (Hu et al., 2021; Liu et al., 2021; Nan et al., 2022). However, few reports on its application in adult sepsis and BSI are available. In this study, we discussed the clinical implications and value of mNGS as a new pathogen detection tool for adult sepsis and BSI.

A total of 194 patients with sepsis and BSI were included in this study. More than half of the patients received mechanical ventilation and vasoactive drug therapy, with high APACHE II and SOFA scores. The four most commonly detected pathogens by mNGS and blood culture method were *Klebsiella pneumoniae*, *Acinetobacter baumannii*, *Escherichia coli*, and *Pseudomonas aeruginosa*, indicating that the common infectious pathogen in ICU is still Gram-negative bacillus, which is consistent with the current prevalence of hospital-related infections.

In a study where mNGS was applied to guide the treatment of 178 patients with severe pneumonia, the patients’ 28-day and 90-day survival rates improved significantly (Xie et al., 2019). A retrospective study of mNGS application in the clinical diagnosis and prognosis of acute respiratory distress syndrome in severe pneumonia showed that the 28-day mortality rate of the mNGS group was significantly lower than that of the non-mNGS group (Zhang et al., 2020). The results from our study, which were consistent with those of (Zhang et al., 2020), showed that the 28-day mortality rate of the mNGS group was significantly lower than that of the non-mNGS group. The total hospitalization time in the mNGS group was longer than that in the non-mNGS group, but the differences in ICU hospitalization time, mechanical ventilation time, vasoactive drug use time, and 90-day mortality rate between the two groups were not statistically significant. This indicated that the patients with sepsis and BSI who underwent blood culture and mNGS are afflicted with the disease for a longer time and have a lower mortality in the short term. This may be due to the earlier adjustment of antibiotic therapy in patients in the mNGS group, which resulted in a higher 28-day survival rate. In contrast, the difference between the 90-day mortality rate in the two groups was not statistically different. Mortality rate is affected by various factors, and further randomized controlled prospective studies are needed to rule out the effect of confounding factors on mortality.

Reports have shown that the sensitivity and positive predictive value of mNGS for bacterial detection are as high as 97.1% and 94.1%, respectively, which is significantly higher than those of traditional bacterial and fungal smears, cultures, inflammatory markers (Fang et al., 2020). A prospective, observational, single-center study of 256 plasma samples from 48 septic patients showed a positive blood culture rate of 11% and a positive mNGS rate of 71% (Grumaz et al., 2019). In a study of critically ill patients suspected of BSI, it was found that the positive rate of mNGS was 68.3% (excluding viruses), and the positive rate of blood culture was 16.7% (Hu et al., 2021). The high positive rate of blood culture in our study and the positive rate of mNGS are comparable to existing literature, which may be related to the fact that 58.8% of the patients were not treated with antibiotics before blood culture.

Pathogens identified by mNGS and blood culture were also compared. The types of bacteria, especially Gram-negative bacilli, detected by blood culture were identical to those of mNGS. The two methods detected the most Gram-negative bacilli, and the sequences were essentially the same, proving that Gram-negative bacilli account for the majority of sepsis and BSI in the ICU. However, in terms of fungi, the blood culture test detected more of the candida species, while the mNGS test detected *Pneumocystis jirovecii* and *Rhizomucor miniaturus*. Relevant research has shown that *Pneumocystis* is almost entirely non-existent in healthy human lungs, but *Pneumocystis* fragments can infiltrate the peripheral blood through respiratory tract infection sites, especially in the case of immunosuppression (Zhang et al., 2019; Geng et al., 2021). Therefore, the detection of fungi commonly found in the lungs by mNGS in peripheral blood has limited value in diagnosing BSI. Low positive rates of mNGS for fungal detection in sepsis patients have been reported (Long et al., 2016; Grumaz et al., 2019). Our study contains similar findings, suggesting that mNGS has limited utility in fungal BSI. Blood cultures failed to detect viruses, unlike mNGS, which not only detected viruses but also detected rare pathogens (*Leishmania donovani* species complex, *Bacillus cereus* population) and was therefore more suitable to detect unknown infections, a finding which is consistent with previous studies (Duan et al., 2021; Geng et al., 2021; Govender et al., 2021; Zhan et al., 2022).

In addition, consistency analysis of mNGS and blood cultures showed that 24.1% of pathogens identified in the mNGS group were consistent with blood cultures, and 75.9% were inconsistent with blood cultures. In the absence of agreement, 77.7% was negative for blood cultures, and 14.1% was negative for mNGS. It indicated that mNGS plays an important role in patients with negative blood cultures and provides a reference for the adjustment of antibiotic therapy in those patients. The use of mNGS for pneumonia pathogen identification was described in a large, multi-center prospective study of 159 patients, resulting in treatment change in 59 patients (37.1%), 40 of which (25.2%) received effective antibiotic treatment while avoiding unnecessary antibiotic use (Zhou et al., 2021). In terms of clinical medication guidance, we found that mNGS has become an important reference for the adjustment of antibiotics in sepsis and BSI in the ICU. However, the adjustment of the first antibiotic treatment according to mNGS did not decrease the severity of the disease in the short term.

The optimal time window for mNGS detection in BSI has yet to be determined due to the lack of studies on the impact of antibiotic therapy on the diagnostic performance of mNGS. This study suggested that the positive rate of mNGS could still be higher than that of culture 1 to 2 weeks after the disease onset (Brenner et al., 2018). Over time, blood culture and mNGS had complementary advantages. Therefore, it is currently recommended that mNGS be performed as soon as possible after the diagnosis of disease in patients. If conditions allow, while blood culture samples are taken and stored at -16 to -20°C, blood samples can be taken for subsequent mNGS. After sub-group analysis, it was found that the total hospitalization time in the late group was longer than that in the early group, and the ICU hospitalization time in the late group was longer than that in the early and intermediate groups, suggesting that the early application of mNGS might shorten the ICU hospitalization time and the total hospitalization time. The 28-day mortality rate was higher in the early group, which might be due to the baseline data. The patients in the early group were older and were in more critical condition. The laboratory results suggested more severe organ damage. Although there was no statistical difference, multiple factors might contribute to the increased mortality rate.

Our study contained a few limitations. Firstly, only the top four pathogens in the number of detection sequences and their relative abundance were considered among the pathogens detected by mNGS. Therefore, other major pathogens might have been missed, resulting in the underestimation of the detected pathogens. Secondly, our research did not study the impact of conducting mNGS on hospital expenses such as hospitalization expenses and ICU hospitalization expenses and the effect it had on the patient’s economic status. Thirdly, propensity score matching was not carried out to rule out confounding variables due to the incomplete comparison of baseline data, which may have had some effect on the mortality rate.

In summary, compared with traditional blood culture methods, mNGS has certain advantages in the diagnosis of pathogens causing BSI and, subsequently, sepsis. In the early stage of sepsis and BSI, simultaneous detection of pathogens using mNGS technology can lead to early adjustments in antibiotic treatment and reduce the mortality rate. It is an advisable early pathogen detection method for sepsis and BSI. The earlier mNGS is performed, the shorter the ICU hospitalization time and the total hospitalization time will be. Future prospective randomized controlled studies with large sampling sizes are needed to comprehensively assess the diagnostic value of mNGS for sepsis and BSI. Moreover, for BSI, the high sequence number and relative abundance of pathogens detected by mNGS as a diagnostic threshold need to be further determined by large-scale clinical studies.

## Data availability statement

The raw data supporting the conclusions of this article will be made available by the authors, without undue reservation.

## Ethics statement

The studies involving human participants were reviewed and approved by The Ethics Committee of Scientific Research and Clinical Trials of the First Affiliated Hospital of Zhengzhou University. Written informed consent for participation was not required for this study in accordance with the national legislation and the institutional requirements.

## Author contributions

YCZ designed the scheme, CQ and SZ were in charge of patient management, FY collected data, and YYZ and XD revised this article. All authors contributed to the article and approved the submitted version.
