# Supplementary material for: Diagnostic value of metagenomic next-generation sequencing in sepsis and bloodstream infection
Source: Front Cell Infect Microbiol. 2023 Feb 10;13:1117987. doi: 10.3389/fcimb.2023.1117987 (PMC9950395; doi:10.3389/fcimb.2023.1117987)
Supplement: Supplementary file 1 [file Table_1.docx]

Supplementary Material

Diagnostic Value of Metagenomic Next-Generation Sequencing in Sepsis and Bloodstream Infection

Cuihong Qin^1†^, Shuguang Zhang^1†^, Yingying Zhao^1^, Xianfei Ding^1^, Fei Yang^1^, Yangchao Zhao^2*^

*** Correspondence:** Yangchao Zhao: zhaoyangchao125@126.com

## Table S1

**Comparison of baseline data between mNGS group and non-mNGS group**

|  | mNGS group  (*n* = 112) | Non-mNGS group (*n* = 82) | *p* |
| --- | --- | --- | --- |
| Male | 80(71.43%) | 48(58.54%) | 0.067 |
| Age (years) | 54.24 ± 15.67 | 57.49 ± 16.04 | 0.160 |
| APACHE Ⅱ | 18.58 ± 8.82 | 20.22 ± 8.24 | 0.190 |
| SOFA | 8.04 ± 4.99 | 8.83 ± 4.18 | 0.243 |
| **Medical history** | | | |
| Diabetes | 29(25.89%) | 25(30.49%) | 0.519 |
| Coronary heart disease | 17(15.18%) | 18(21.95%) | 0.259 |
| Chronic kidney disease | 8(7.14%) | 7(8.54%) | 0.789 |
| Immunosuppression | 7(6.25%) | 9(10.98%) | 0.294 |
| Chronic lung disease | 3(2.68%) | 1(1.22%) | 0.639 |
| Cerebral apoplexy | 18(16.07%) | 26(31.71%) | 0.013 |
| Hypertension | 44(39.29%) | 39(47.56%) | 0.254 |
| Chronic liver disease | 7(6.25%) | 6(7.32%) | 0.773 |
| **Biochemical indicators** |  | | |
| White blood cell | 11.72 ± 11.99 | 11.94 ± 8.06 | 0.884 |
| Red blood cell | 3.32 ± 0.92 | 3.07 ± 0.94 | 0.073 |
| Hemoglobin | 100.49 ± 27.28 | 92.43 ± 27.28 | 0.048 |
| Platelet | 154.93 ± 137.89 | 128.72 ± 119.25 | 0.170 |
| Procalcitonin | 11.90 ± 26.05 | 9.95 ±15.46 | 0.549 |
| C-reactive protein | 131.11 ± 118.36 | 148.13 ± 96.17 | 0.290 |
| Erythrocyte sedimentation rate | 60.61 ± 37.71 | 71.27 ± 42.67 | 0.482 |
| Interleukin-6 | 810.76 ± 1760.98 | 890.13 ± 1903.76 | 0.868 |
| Serum creatinine | 148.12 ± 147.11 | 161.58 ± 196.95 | 0.586 |
| Blood urea nitrogen | 12.99 ± 8.87 | 14.57 ± 12.94 | 0.315 |
| Alanine aminotransferase | 105.46 ± 213.14 | 132.84 ± 607.37 | 0.659 |
| Aspartate transaminase | 161.05 ± 407.15 | 145.95 ± 774.95 | 0.861 |
| Total bilirubin | 29.44 ± 40.76 | 35.92 ± 51.19 | 0.328 |
| Albumin | 29.31 ±7.26 | 28.52 ± 6.63 | 0.440 |
| Prothrombin time | 16.89 ± 15.40 | 15.49 ± 4.70 | 0.428 |
| Activated partial thromboplastin time | 35.76 ± 14.86 | 34.45 ± 11.34 | 0.506 |
| International standardized ratio | 1.54 ± 1.56 | 1.38 ± 0.42 | 0.383 |

## Table S2

**Comparison of baseline data of early, intermediate, and late groups**

|  | Early group (*n* = 47) | Intermediate group (*n* = 35) | Late group (*n* = 30) | *p* |
| --- | --- | --- | --- | --- |
| Male | 32(68.01%) | 28(80%) | 20(66.67%) | 0.396 |
| Age (years) | 56.40 ± 16.16 | 53.23 ± 15.76 | 52.03 ± 14.84 | 0.445 |
| APACHE Ⅱ | 20.53 ± 9.78 | 16.89 ± 7.64 | 17.50 ± 8.18 | 0.132 |
| SOFA | 8.66 ± 6.33 | 7.69 ± 3.47 | 7.47 ± 4.03 | 0.526 |
| **Medical history** | | | | |
| Diabetes | 13(27.66%) | 10(28.57%) | 6(20.00%) | 0.687 |
| Coronary heart disease | 6(12.77%) | 4(11.43%) | 7(23.33%) | 0.342 |
| Chronic kidney disease | 2(4.26%) | 3(8.57%) | 3(10.00%) | 0.586 |
| Immunosuppression | 1(2.13%) | 4(11.43%) | 2(6.67%) | 0.226 |
| Chronic lung disease | 22(4.26%) | 0(0.0%) | 1(3.33%) | 0.482 |
| Cerebral apoplexy | 5(10.64%) | 5(14.29%) | 8(26.67%) | 0.165 |
| Hypertension | 16(34.04%) | 15(42.86%) | 13(43.33%) | 0.627 |
| Chronic liver disease | 4(8.51%) | 3(8.57%) | 0(0.00%) | 0.55 |
| **Biochemical indicators** | | | | |
| White blood cell | 15.12 ± 14.03 | 8.09 ± 10.08 | 10.61 ± 9.13 | 0.231 |
| Red blood cell | 3.37 ± 1.05 | 3.13 ± 0.73 | 3.45 ± 0.88 | 0.213 |
| Hemoglobin | 101.23 ± 31.86 | 95.09 ± 21.08 | 105.65 ± 25.57 | 0.291 |
| Platelet | 180.45 ± 145.92 | 145.00 ± 144.68 | 126.53 ± 111.59 | 0.350 |
| Procalcitonin | 11.89 ± 22.12 | 9.72 ±23.17 | 14.47 ± 34.17 | 0.217 |
| C-reactive protein | 122.85 ± 110.08 | 134.73 ± 120.5 | 138.99 ± 130.83 | 0.577 |
| Erythrocyte sedimentation rate | 65.67 ± 42.10 | 52.45 ± 40.84 | 64.80 ± 18.75 | 0.121 |
| Interleukin-6 | 1243.71 ± 2271.16 | 549.18 ± 1242.64 | 135.21 ± 135.66 | 0.115 |
| Serum creatinine | 160.99 ± 146.05 | 125.95 ± 130.86 | 153.83 ± 167.42 | 0.619 |
| Blood urea nitrogen | 14.60 ± 9.20 | 11.51 ± 8.85 | 12.19 ± 8.21 | 0.865 |
| Alanine aminotransferase | 126.72 ± 212.35 | 89.40 ± 211.67 | 90.87 ± 220.35 | 0.684 |
| Aspartate transaminase | 190.00 ± 334.14 | 128.51 ± 297.08 | 153.67 ± 592.67 | 0.843 |
| Total bilirubin | 30.56 ± 41.74 | 34.64 ± 50.82 | 21.62 ± 21.67 | 0.412 |
| Albumin | 29.27 ± 6.31 | 28.04 ± 8.63 | 30.83 ± 6.84 | 0.113 |
| Prothrombin time | 19.37 ± 22.70 | 15.15 ± 4.76 | 15.01 ± 6.84 | 0.482 |
| Activated partial thromboplastin time | 39.36 ± 20.09 | 32.40 ± 7.40 | 34.03 ± 10.18 | 0.151 |
| International standardized ratio | 1.79 ± 2.32 | 1.37 ± 0.43 | 1.35 ± 0.63 | 0.437 |
